# Supplementary material for: Resolving the full spectrum of human genome variation using Linked-Reads
Source: Genome Res. 2019 Apr;29(4):635–45. doi: 10.1101/gr.234443.118 (PMC6442396; doi:10.1101/gr.234443.118)
Supplement: Supplemental Material [file supp_gr.234443.118_Supplemental_longranger-2.2.zip › longranger-2.2.tar.bz2!/lr2.2-public/lib/go/src/code.google.com/vitess/docs/user-guide/schema-management.html]

Vitess / Schema Management


Toggle navigation

- Overview
- Guides
- Reference
- Blog
- GitHub

- #### Overview

  - What is Vitess
  - Scaling MySQL with Vitess
  - Key Concepts
- #### Getting Started

  - Run Vitess on Kubernetes
    - Custom Docker Build
  - Run Vitess Locally
- #### User Guide

  - Introduction- Client Libraries- Backing Up Data- Reparenting
        - Schema Management
        - VSchema Guide
        - Schema Swap (Tutorial)
        - Sharding
          - Horizontal Sharding (Tutorial, automated)
          - Horizontal Sharding (Tutorial, manual)
          - Sharding in Kubernetes (Tutorial, automated)
          - Sharding in Kubernetes (Tutorial, manual)
        - Vitess Sequences
        - MySQL Server Protocol
        - Vitess and Replication
        - Update Stream
        - Row Based Replication
        - Topology Service
        - Transport Security Model
        - Launching
          - Scalability Philosophy
          - Production Planning
          - Server Configuration
          - 2PC Guide
          - Troubleshooting
        - Upgrading
- #### Reference Guides

  - Vitess API- vtctl Commands
- #### Other Resources

  - Presentations- Blog- Roadmap
- #### Contributing

  - Contributing to Vitess- GitHub Workflow- Code Reviews
- GitHub

# Schema Management

- #### Overview

  - What is Vitess
  - Scaling MySQL with Vitess
  - Key Concepts
- #### Getting Started

  - Run Vitess on Kubernetes
    - Custom Docker Build
  - Run Vitess Locally
- #### User Guide

  - Introduction- Client Libraries- Backing Up Data- Reparenting
        - Schema Management
        - VSchema Guide
        - Schema Swap (Tutorial)
        - Sharding
          - Horizontal Sharding (Tutorial, automated)
          - Horizontal Sharding (Tutorial, manual)
          - Sharding in Kubernetes (Tutorial, automated)
          - Sharding in Kubernetes (Tutorial, manual)
        - Vitess Sequences
        - MySQL Server Protocol
        - Vitess and Replication
        - Update Stream
        - Row Based Replication
        - Topology Service
        - Transport Security Model
        - Launching
          - Scalability Philosophy
          - Production Planning
          - Server Configuration
          - 2PC Guide
          - Troubleshooting
        - Upgrading
- #### Reference Guides

  - Vitess API- vtctl Commands
- #### Other Resources

  - Presentations- Blog- Roadmap
- #### Contributing

  - Contributing to Vitess- GitHub Workflow- Code Reviews

Your MySQL database schema lists the tables in your database and
contains table definitions that explain how to create those tables.
Table definitions identify table names, column names, column types,
primary key information, and so forth.

This document describes the `vtctl`
commands that you can use to review or
update your schema in Vitess.

Note that this functionality is not recommended for long-running schema changes. In such cases, we recommend to do a schema swap instead.

## Reviewing your schema

This section describes the following `vtctl` commands, which let you look at the schema and validate its consistency across tablets or shards:

- GetSchema
- ValidateSchemaShard
- ValidateSchemaKeyspace

### GetSchema

The `GetSchema` command
displays the full schema for a tablet or a subset of the tablet's tables.
When you call `GetSchema`, you specify the tablet alias that
uniquely identifies the tablet. The `<tablet alias>`
argument value has the format `<cell name>-<uid>`.

**Note:** You can use the
`vtctl ListAllTablets`
command to retrieve a list of tablets in a cell and their unique IDs.

The following example retrieves the schema for the tablet with the
unique ID `test-000000100`:

```
GetSchema test-000000100
```

### ValidateSchemaShard

The
`ValidateSchemaShard`
command confirms that for a given keyspace, all of the slave tablets
in a specified shard have the same schema as the master tablet in that
shard. When you call `ValidateSchemaShard`, you specify both
the keyspace and the shard that you are validating.

The following command confirms that the master and slave tablets in
shard `0` all have the same schema for the `user`
keyspace:

```
ValidateSchemaShard user/0
```

### ValidateSchemaKeyspace

The `ValidateSchemaKeyspace`
command confirms that all of the tablets in a given keyspace have
the the same schema as the master tablet on shard `0`
in that keyspace. Thus, whereas the `ValidateSchemaShard`
command confirms the consistency of the schema on tablets within a shard
for a given keyspace, `ValidateSchemaKeyspace` confirms the
consistency across all tablets in all shards for that keyspace.

The following command confirms that all tablets in all shards have the
same schema as the master tablet in shard `0` for the
`user` keyspace:

```
ValidateSchemaKeyspace user
```

## Changing your schema

This section describes the `vtctl ApplySchema` command, which
supports schema modifications. Vitess' schema modification functionality
is designed the following goals in mind:

- Enable simple updates that propagate to your entire fleet of servers.
- Require minimal human interaction.
- Minimize errors by testing changes against a temporary database.
- Guarantee very little downtime (or no downtime) for most schema updates.
- Do not store permanent schema data in the topology server.

Note that, at this time, Vitess only supports
data definition statements
that create, modify, or delete database tables.
For instance, `ApplySchema` does not affect stored procedures
or grants.

### ApplySchema

The `ApplySchema`
command applies a schema change to the specified keyspace on every
master tablet, running in parallel on all shards. Changes are then
propagated to slaves via replication. The command format is:
 `ApplySchema {-sql=<sql> || -sql_file=<filename>} <keyspace>`

When the `ApplySchema` action actually applies a schema
change to the specified keyspace, it performs the following steps:

1. It finds shards that belong to the keyspace, including newly added
   shards if a resharding event
   has taken place.
2. It validates the SQL syntax and determines the impact of the schema
   change. If the scope of the change is too large, Vitess rejects it.
   See the permitted schema changes section
   for more detail.
3. It employs a pre-flight check to ensure that a schema update will
   succeed before the change is actually applied to the live database.
   In this stage, Vitess copies the current schema into a temporary
   database, applies the change there to validate it, and retrieves
   the resulting schema. By doing so, Vitess verifies that the change
   succeeds without actually touching live database tables.
4. It applies the SQL command on the master tablet in each shard.

The following sample command applies the SQL in the **user\_table.sql**
file to the **user** keyspace:

```
ApplySchema -sql_file=user_table.sql user
```

#### Permitted schema changes

The `ApplySchema` command supports a limited set of DDL
statements. In addition, Vitess rejects some schema changes because
large changes can slow replication and may reduce the availability
of your overall system.

The following list identifies types of DDL statements that Vitess
supports:

- `CREATE TABLE`
- `CREATE INDEX`
- `CREATE VIEW`
- `ALTER TABLE`
- `ALTER VIEW`
- `RENAME TABLE`
- `DROP TABLE`
- `DROP INDEX`
- `DROP VIEW`

In addition, Vitess applies the following rules when assessing the
impact of a potential change:

- `DROP` statements are always allowed, regardless of the
  table's size.
- `ALTER` statements are only allowed if the table on the
  shard's master tablet has 100,000 rows or less.
- For all other statements, the table on the shard's master tablet
  must have 2 million rows or less.

If a schema change gets rejected because it affects too many rows, you can specify the flag `-allow_long_unavailability` to tell `ApplySchema` to skip this check.
However, we do not recommend this. Instead, you should apply large schema changes by following the schema swap process.

Contact: vitess@googlegroups.com  **·**  
Announcements  **·**  
© 2017 Vitess powered by Google Inc
